# A psycho-social-environmental lens on radon air pollutant: authorities’, mitigation contractors’, and residents’ perceptions of barriers and facilitators to domestic radon mitigation

**DOI:** 10.3389/fpubh.2023.1252804

**Published:** 2023-08-15

**Authors:** David Hevey, Tanja Perko, Meritxell Martell, Gary Bradley, Sofie Apers, Kateřina Navrátilová Rovenská

**Affiliations:** ^1^School of Psychology, Trinity College Dublin, Dublin, Ireland; ^2^SCK CEN, Institute for Environment, Health and Safety, Belgian Nuclear Research Centre, Mol, Belgium; ^3^Department of Political Science, University of Antwerp, Antwerp, Belgium; ^4^Merience SCP, Barcelona, Spain; ^5^Department of Communication Studies, University of Antwerp, Antwerp, Belgium; ^6^National Radiation Protection Institute (SURO), Prague, Czechia

**Keywords:** radon, lung cancer, risk, mitigation, barriers, facilitators

## Abstract

**Introduction:**

Radon is a major indoor air pollutant that poses a significant risk of lung cancer to those exposed in their homes. While mitigation of high radon levels in homes has been shown to be effective, home mitigation rates remain low. This study examines the barriers and facilitators to radon mitigation in homes from the perspectives of authorities responsible for radon risk management, the mitigation industry (contractors), and residents in four European countries (Belgium, Ireland, Slovenia, and the UK) with high radon risks and low mitigation rates.

**Methods:**

A multi-method approach was used to gather data from various stakeholders, including online surveys, content analysis of legal documents, group interviews, workshops, and focus groups.

**Results:**

Authorities, contractors, and residents identified various facilitators to radon mitigation, including legal requirements for mitigation, awareness campaigns, low mitigation costs, availability of financial support, accreditation of mitigation contractors, and a perception of radon as a health threat. However, barriers to mitigation were also identified, such as a lack of awareness, fragmented mitigation processes, and inadequate communication between stakeholders.

**Discussion:**

The study highlights the complexity of the radon mitigation process and suggests that interventions aimed at increasing mitigation rates should target stakeholders beyond just residents, such as constructors, health professionals, and policy makers. An integrated approach to radon mitigation, from policy to provision, is necessary to effectively lower levels of this indoor air pollutant.

## Introduction

Radon is a naturally occurring radioactive gas that can accumulate in indoor environments and is one of the major indoor air pollutants (1). According to the World Health Organization (WHO), radon is one of the leading causes of lung cancer: it is estimated that it causes around 21,000 deaths annually in the European Union alone (2). Although radon exposure in the home increases the risk of lung cancer, this risk can be managed by testing and mitigation. Unfortunately, low mitigation rates of dwellings with high radon risks are reported in all countries in Europe (3, 4) and worldwide (5–7) indicating a value-action gap (8). The value action gap is a well-established phenomenon, where attitudes about protecting one’s health do not result in the protective behaviour being performed.

The Council Directive 2013/59/Euratom Basic Safety Standards (9) mandates that European Union Member States (EU MS) establish National Radon Action Plans (RAPs) to reduce radon exposure and ultimately the risk of lung cancer. Annex XVIII of the Directive provides a list of items to be considered by authorities in preparing a RAP. RAPs should include measures such as radon mapping, the promotion of radon resistant construction techniques to prevent radon ingress into new buildings and the provision of information to the public on radon risks and mitigation measures. Each EU MS should establish its own national reference levels for the annual average concentration of radon in air in indoor workplaces and dwellings. National reference level shall not be higher than 300 Bq/m^3^ radon concentration for both, dwellings and workplaces (9); however, in some countries, for instance Ireland, the reference level is 200 Bq/m^3^. The WHO recommends that countries adopt reference levels of the radon gas of 100 Bq/m^3^. If this level cannot be implemented under the prevailing country-specific conditions, the WHO recommends that the reference level should not exceed 300 Bq/m^3^ (2).

Where radon levels in buildings exceed the reference level, mitigation can be performed to the building. However, evidence consistently indicates that testing for radon and subsequent home mitigation rates are generally low in many countries, even if indoor radon concentrations can be cost-effectively reduced (10). It is worth noting that existing research has primarily focused on the impact of radon awareness campaigns and risk perception on increased radon testing (11, 12), with fewer studies of interventions reporting on indoor radon mitigation (13, 14), overlooking value-action gap related to mitigation. There is a need to better understand the factors that hinder and facilitate radon mitigation after receiving a high indoor radon test result (15). However, in order to develop such an understanding, it is essential that a broad perspective is taken.

The successful mitigation of radon in dwellings requires coordination and cooperation among multiple stakeholders, including authorities, the mitigation industry, and residents or owners (16–18). To date, research has primarily examined the radon mitigation from the perspective of the residents but has neglected the role that other stakeholders play in facilitating mitigation. Legal authorities are responsible for regulation of the indoor radon exposure and the implementation of national strategies (9). The mitigation industry conducts the technical work to reduce radon levels in existing buildings. Residents or owners of domestic buildings are responsible for testing for radon concentrations and mitigating the dwellings if exceeded rates of radon detected (19).

Research (20–24) worldwide shows that during the past decade an important component of radon control strategies directed at the public has been to educate, inform, encourage, persuade and engage the public to measure radon in their homes and to mitigate the dwelling if considered necessary; however the “*voluntary public responses to these efforts have been disappointingly low*” (3).

Radon awareness campaigns typically used fear appeal and technical knowledge about radon (23) to target individuals to encourage them to test and, where appropriate, mitigate. Although some research focuses lack of knowledge of radon as the main issue to address, simply increasing awareness, knowledge or risk perception does not necessarily improve risk mitigation behaviours (9, 25). Education alone is not sufficient for increasing health-protective behavioural action (26, 27); people are complex and have factors that interact, influencing decisions on preventive action (28). Consequently research needs to examine the lack of mitigation action from a more comprehensive psycho-social-environmental lens. A broad range of behavioural (e.g., perceived lack of time), psychological (e.g., self-efficacy), educational (e.g., low level of educational attainment), socio-economic (e.g., low income) and social factors (e.g., lack of home mitigation performed others in similar high radon risk area) may influence radon mitigation rates (6, 29–34). However, focusing only on the individuals neglects the context in which the individual acts and the critical role that other people and organisations/agencies in their environment have on decision-making by residents regarding radon. The individual’s environment comprises of important others in the community (e.g., healthcare professionals) who may encourage radon mitigation, as well as the availability of those providing radon mitigation services (e.g., contractors). In addition, the policies and practices of radon responsible authorities at a national or state-level will also impact on the individual’s decision options and consequently mitigation rates.

The objective of this paper is to shed light on the critical issue of successful mitigation of radon in dwellings by examining the barriers and facilitators from the perspectives of key stakeholders from various levels: responsible authorities, the mitigation industry (contractors) and the residents. The study takes place in four countries with high radon risks and low mitigation rates, namely Belgium, Ireland, Slovenia and the United Kingdom (UK), with similar radon risks and diverse approaches to radon risk management in a period between 2022 and 2023, during a period of intensive implementation of a new legislation in the four countries (35) (see Table 1 for key comparisons).

**Table 1:** Relevant indicators for radon mitigation in Belgium, Ireland, Slovenia and the United Kingdom.

	Belgium	Ireland	Slovenia	United Kingdom
Estimated lung cancer deaths due to radon per year (country population)	440 (11.6 million inhabitants)^a^	350 (5 million inhabitants)^b^	60 (2 million inhabitants)^c^	1,100 (6,733 million inhabitants)^d^
Reference levels for dwellings	300 Bq/m^3^	200 Bq/m^3^	300 Bq/m^3^	200 Bq/m^3^
Supporting levels for dwellings	Target level of 100 Bq/m^3^ intervention level of 600 Bq/m^3^	—	—	Target level of 100 Bq/m^3^
Level of radon awareness among residents in radon risk areas (%)^d^	75% (*N* = 300)	59% (*N* = 1,003 in the entire territory)	75% (*N* = 453)	—
Objectives of mitigation in the national radon action plan	Mitigate all buildings with a radon concentration above and around the reference level (approx. 36,000 dwellings affected)	Mitigation rate increased to 30% for all homes over the reference level where financial support is available to householders by 2024. At present mitigation rate is about 20%	There is no overview of number of dwellings needed to be mitigated; nor is any estimate of need available	—
Advice on radon protection in building code	Yes (regional competence). The regional construction code of the radon priority area impose the description of the radon protective measures by the responsible architect in the building permit	All dwellings built after 1998 in high radon areas must be fitted with radon preventive measures, namely a radon membrane and a standby radon sump. The standby sump can be activated if high radon concentrations are found in the building	Yes (guideline for construction of radon safe new buildings)	Building regulations require that buildings in certain areas should be constructed with protective measures that aim to prevent radon ingress
Registration of radon mitigation contractors	Registration is based on following a specific training on radon and being active in the radon field. Invited to repeat the training course every year, but not obligatory	Registration consists of participating successfully in a radon remediation course, tax compliance and public liability insurance. To maintain registration, the contractor has to report annually anonymised data in relation to the provision of radon remediation services to EPA	No registration is established yet	No particular licensing for radon mitigators is in place (only a building contractor licensed for electrical work). There are courses available by different organisations but no need to be accredited
Information on available mitigation contractors	Published at responsible authority’s website	Published on responsible authority’s website	Not available on official website	Radon Council and the UK Radon Association publish lists of contractors provided by their members
Requirements on conveyancing	—	Three questions on radon testing are included in the conveyancing process that alert the prospective buyer to the risk of radon	—	During the house buying and selling process, radon is included in the conveyancing process through the local authority search and property information form completed by the seller
Links to indoor air quality (IAQ) programmes	In some cases, radon is included in the indoor air quality measurements	The UNVEIL project aims to understand ventilation and radon in energy efficient buildings^f^	Radon included in indoor air quality measurements when energy-saving interventions such as energy renovations and window replacements are carried out	There is regular interaction and cross-fertilisation of ideas between organisations working on radon and those engaged in air quality. Trainings are connected with trainings on indoor air quality by the Chartered Institution of Building Services Engineers (CIBSE) and other professional organisations. Radon is part of the indoor air quality programme: legislation on ventilation has been updated https://www.gov.uk/government/publications/building-regulations-approved-documents-l-f-and-overheating-consultation-version
Links to energy saving programmes	—	Radon is a recommendation included in building energy rating reports for home energy performance	It is advised to measure during energy saving interventions but not required	Large survey looking into radon concentrations in houses in 1990s and recently investigating energy saving measures put in houses demonstrate that radon concentrations have gone up. In the near future, radon is expected to become part of energy saving programmes
Average estimated costs of mitigation of a standard dwelling	500–5,000 €	1,000 € financial support for older adult people (over 65) for remediation is available—there are a range of criteria to be met in order to qualify which are not specific for radon. Additional actions for a financial support are on-going	1,000–10,000 €	£800–£2,000, and £5,000 if it is a complicated remediation. In case of low incomes, support is provided in the form of local authorities’ grants

a Data from FANC website in 2016 (7% of lung cancer due to radon). https://afcn.fgov.be/fr/dossiers/radon-et-radioactivite-dans-votre-habitation/radon/quel-est-le-risque-pour-votre-sante.

b EPA Ireland. (2022). Radon in homes. https://www.epa.ie/publications/monitoring--assessment/radon/EPARadonHome2022.pdf.

c Birk M, Žagar, T, Tomšič S, Lokar, K, Mihor A, Bric, N, Mlakar M, and Zadnik V (2022). Impact of radon on lung cancer incidence in Slovenia. Onkologija. 26. 16–21. https://doi.org/10.25670/oi2022-008on.

d Public Health England. (2018). UK National Radon Action Plan. PHE-CRCE-043.

e The question was included in a representative public opinion survey in the three countries analysed in 2023 as part of the RadoNorm European project. The formulation of the question was: “do you know anything about radon?” and the answer categories included: “no,” “I have heard something about it” and “yes.” The percentage considered under the “level of radon awareness among residents” includes the answers “I have heard something about it” and “yes.” Source: RadoNorm pilot study report from public opinion survey Belgium 2020 2021. Development of a modular questionnaire for investigating societal aspects of radon and NORM. Doi: 10.20348/STOREDB/1174/1251.

f McGrath JA and Byrne MA. (2015). UNVEIL: Understanding Ventilatino and radon in energy efficient buildings in Ireland. EPA research report by National University of Ireland Galway. https://www.epa.ie/publications/research/environment--health/Research_Report_273.pdf.

This study is the first of its kind to investigate these stakeholders and countries simultaneously and aims to provide empirical evidence to support policy and decision-making processes in the implementation of new legislation towards improved indoor air quality. By understanding the challenges and opportunities faced by each group, it may be possible to develop more effective strategies for reducing radon exposure and achieving compliance with legal limits.

## Materials and methods

A multi method approach was used to obtain data from each of the key stakeholders. Ethical approval for this study in Ireland and the UK was obtained from Trinity College, Dublin (SPREC092021-04), and for the focus groups in Belgium and Slovenia ethical approval was obtained through the University of Antwerp (no. SHW_21_135).

### Responsible authorities’ perspectives

The study “*Review and evaluation of national radon action plans established in EU Member States according to the requirements in Council Directive 2013/59/Euratom*” investigated, among other aspects, the authorities’ perspectives on barriers and facilitators for radon mitigation. A mixed method approach was used, comprising an on-line survey, content analysis of legal documents, group interviews, four regional workshops and the final workshop. The study was conducted in 27 EU MS and the UK. Firstly, the on-line survey allowed the authors to identify responsible authorities for different aspects of radon management including radon mitigation. Secondly, for the legal document review and content analysis, the authors collected RAPs or related legal documents if RAPs were not yet developed. A protocol for the analysis guided the extraction of data to be analysed. Coders received specific training to ensure that the same method was used when analysing the data. The mitigation aspects were coded by two independent coders. In case of different data extracted and different codes, the third (master) coder discussed the differences and decided on the final data extracted (agreed data). Thirdly, the findings from the legal document content analysis were discussed with representatives in each country through group interviews. These group interviews (between 2 and 15 people) were conducted to validate the information as well as to respond on and clarify any missing information related to mitigation. The guiding questions for the interviews included: “*Does the RAP define or include information regarding a strategy for post construction mitigation?*”; “*Which are procedures facilitating mitigations?*”; “*Has the EU MS* (*or the UK*) *implemented existing policy/policies for facilitating mitigations?*”; “*Are there any existing methods and tools* (e.g.*, building code*) *for facilitating mitigations?*”; and “*Are there considered any indicators to measure the effectiveness of mitigations?*” Notes from the interviews were sent back to all representatives for verification and additional feedback. Group interviews were conducted on-line between October 2021 and March 2022. In addition, four regional workshops were held to examine similarities and differences in mitigation actions, barriers and facilitators for mitigation of dwellings. The main focal points at the workshops included: (*a*) *How can authorities ensure a significant difference in radon exposure through mitigation?* and (*b*) *How to motivate industry to offer mitigation services?* The four workshops were conducted between October 2021 and March 2022. Finally, the overall results were discussed, verified, and compared at a workshop in Brussels, Belgium, with 50 participants from various countries in September 2022. The workshop covered topics such as procedures for facilitating mitigations, support for mitigation, guidelines for mitigation, and challenges related to mitigation and approaches to overcome them. For this paper, the general findings of the study have been substantiated with the specific findings in Belgium, Ireland, Slovenia and the UK.

### Mitigation industry (contractor’s) perspectives

A protocol for semi-structured interviews was derived from existing research evidence on radon, as well as self-determination theory (SDT) (36) and entrepreneurship. SDT was used as a guiding framework to design the protocol for eliciting views regarding company history, level of mitigation work carried out, motivation, advertising and customer contact.

Interviewees were drawn from the Environmental Protection Agency (EPA) of Ireland and UK Radon Association (UKRA) lists of registered contractors. We selected Ireland and the UK as both countries have well-established regulatory oversight of radon mitigation and have dedicated radon agencies to provide easily accessible lists of contractors to members of the public seeking remediation. From a total of 25 contractors listed, 5 did not offer domestic radon mitigation services and were excluded. Of the remaining 20, 6 (*n* = 3 Irish and *n* = 3 UK) contractors agreed to interview who currently deliver domestic radon mitigation services. Six refused to interview, and 9 did not return calls.

Potential interviewees were initially contacted by email and follow-up telephone call. All participants were advised that interviews were confidential and their rights under General Data Protection Regulation (GDPR) were explained, and reporting would safeguard anonymity. Verbal consent was requested at the start of the interviews, which took place between October 2022 and January 2023. Semi-structured interviews were recorded remotely using MS Teams. Transcriptions were generated automatically and edited manually to improve readability. NVivo was used for content and thematic analysis (37).

### Residents’ perspectives

Data for gathering residents perspectives were collected from focus groups comprised of individuals living in dwellings with radon levels exceeding the national reference level in Belgium, Ireland and Slovenia. These focus groups were conducted in radon-priority areas in the three countries, which share similar radon risks but differ in their approach to radon risk communication. Table 1 outlines the key features of the selected countries in terms of radon, legal regulations and mitigation service provision. All three countries have strategies in place for raising awareness about radon, but these strategies vary. In Belgium, awareness is primarily raised through limited use of social media and press articles, and the effectiveness of these campaigns is evaluated to some extent. In Slovenia, the focus is more on providing personal advice and responding to radon-related questions, but the effectiveness of radon awareness campaigns is not evaluated. In contrast, Ireland has an evidence-based, theory-based and strategic approach to radon risk communication and also supports citizen science projects related to radon testing and mitigation. The countries were selected as the differences reflect the variation that exists across the EU and will provide insight into commonly experienced barriers for mitigation across different contexts, as well as allowing for unique national experiences to be considered.

For the focus group analysis, each of the focus groups’ discussion was transcribed and thematic analysis was applied (38). Transcripts were reviewed in detailed for familiarity and accuracy before coding. Initial coding was performed in NVivo through multiple readings of the transcript. Following review of the codes, data were coded as barriers or facilitators of mitigation. Each focus group was initially coded separately, then combined for the final analysis.

#### Belgium

Two focus groups were conducted in the context of a broader study “*Co-Designing Communication: A Design Thinking Approach Applied to Radon Health Communication*,” in which the focus was on discovering barriers and facilitators of mitigation behaviour (39). Participants in these focus groups had tested their homes and were recruited for this study through local authorities. The Belgian focus groups (*n* = 5) took place in March 2022 and were held online due to Covid-restrictions in place at the time. Both focus groups lasted around 2 h each. In the first part of the focus groups, barriers were identified that participants experienced throughout their mitigating process. Participants were encouraged to identify as many issues that they had experienced as they could think of, which were subsequently discussed in the group. In the second part of the focus groups, participants evaluated all the barriers and voted for those they found most important.

#### Ireland

Two on-line focus groups were conducted in January–February, 2022. The first group consisted of six people who did not mitigate their dwelling while the second group consisted of four participants who had mitigated. All participants received indoor radon test levels above 200 Bq/m^3^ and are from high radon priority areas. Most participants were homeowners, and only one participant rented their accommodation. Participants were recruited through the Environmental Protection Agency Ireland (EPA). Each focus group had a brief introduction, followed by a series of open-ended questions related to perceived barriers and facilitators to indoor radon mitigation. Participants reviewed the key barriers and facilitators and were asked for their perspective on which barriers or facilitators had the greatest impact on indoor radon mitigation.

#### Slovenia

Similar to Belgium, two focus groups were conducted in the context of the broader study “*Co-Designing Communication: A Design Thinking Approach Applied to Radon Health Communication*.” Participants had tested their homes and were recruited through local authorities. The Slovenian focus group (*n* = 9) took place in May 2022 and was held face-to-face. Both focus groups lasted around 2 h each and followed the same protocol as the Belgian focus groups.

## Results

Results are presented by stakeholder group, discussing the main barriers and facilitators for each group.

### Authorities’ perspective

The key issues raised relate to the legal (requirements for mitigation) and policy (e.g., the connection with indoor air quality and energy saving programmes) issues, communication and awareness campaigns, and the mitigation process (the cost and availability of financial support for mitigation, accreditation of radon mitigation contractors; education and training, and guidelines/instructions on corrective actions).

#### Legal requirements to mitigate dwellings with an exceeded radon concentration

In the countries analysed, the mitigation of private dwellings is not legally required but only advised. The lack of a legal mandate requiring the owner of the residence to mitigate is a barrier for radon mitigation.

#### Connection with indoor air quality and energy saving programmes

National RAPs may be linked with related programmes, such as energy saving and indoor air quality (IAQ). However, in general there are no systematic links with these programmes. Firstly, there are many attempts to connect energy saving programmes with radon prevention programmes; however, these are not systematic. Secondly, links with IAQ programmes are rather scarce and weak. In Belgium, only in some cases radon is included in IAQ measurements. In Slovenia, IAQ should include radon concentrations when energy-saving interventions are performed on public buildings in radon priority areas. The advisory guidance for contractors and householders from the Sustainable Energy Authority of Ireland on more energy efficient homes includes radon.^1^ These connections are recognised as important facilitators for mitigation.

There is a need to further explore the counter effects of implementing energy efficiency measures compared to the increasing exposure to radon. The UK authorities referred to studies that indicate that increasing the airtightness of English homes, without providing compensatory ventilation, would increase indoor radon concentrations by around 60%, resulting in 278 deaths per year (40, 41).

During the study to review RAPs, the authorities discussed how to consider radon in relevant corresponding programmes, particularly for energy saving and IAQ programmes. Despite the lack of human resources in authorities to promote these connections, the benefits of establishing links different authorities were recognised as facilitators. Contacting energy agencies dealing with energy efficiency to raise awareness of the counter effects of implementing energy efficiency measures compared to the increasing exposure to radon was proposed. Furthermore, the connection of radon programmes with energy saving programmes at European level, such as the European Green Deal, was regarded as a facilitator.

#### Communication and awareness campaigns

Authorities organise communication and awareness campaigns annually within radon-priority areas in order to inform citizens about the risk of the indoor air pollutant radon and to motivate citizens living in a dwelling with radon concentration above the reference level to undertake mitigation actions. A wide range of groups are targeted by these communication campaigns, for instance, trade unions, practitioners, family doctors and lung cancer specialists in Belgium; building professionals, owners, sellers, local authorities and health and safety professionals in Ireland; local decision-makers and the general public in Slovenia and solicitors, purchasers and the law society in the UK. However, the lack of evidence-based, strategic and theory-founded communication campaigns is another barrier for increasing mitigation.

#### Costs and financial support for mitigation

Mitigation may range from €500 to more than €5,000 for private dwellings. This expense is usually a burden for residents and the owners of dwellings. Financial support for mitigation is provided in Belgium by the regional government. In Ireland, financial support was provided in a high radon area in which free radon testing was offered in conjunction with a 50% grant toward necessary remedial work. The empirical research conducted in 2018 by the Environmental Protection Agency (EPA) Ireland showed a very low uptake of grants from the citizens (42). In the UK, the Health Security Agency maintains an ongoing programme of work to promote action on radon and encouraging remediation in homes, using resources from national government where these are available.

#### Availability of radon mitigation contractors

Representatives of authorities at the workshops see the prevailing low interest of the building sector in providing radon mitigation services as a significant barrier to increase mitigation rates. They identified a low profit caused by low interest in mitigation as the main reason for the lack of motivation to provide mitigation services, especially for private dwellings.

#### Accreditation of radon mitigation contractors

Although accreditation or license for mitigation can facilitate the uptake of mitigation, the reality is that accreditations or licenses for mitigation are not issued in the majority of the EU MS countries. An exception is Ireland, where radon mitigation contractors are registered by authorities. For this, the contractor has to participate in radon mitigation training, prove tax compliance and provide liability insurance. The contractor has to report annually anonymised information on how many, where and what type of mitigation has been done. In return, a list of registered contractors for mitigation works is published on the internet site of the Irish authorities. In Belgium, remediators are advised to take a training course and are invited to repeat the training course every year, but they are not obliged. The Belgian authorities publish a list of contractors for mitigation of dwellings on their internet pages, but they do not guarantee the quality of the mitigation conducted by those contractors.

#### Education and training

Training courses, seminars or lectures for building professionals and workers in the building industry are or will be organised in most countries. A certificate of attendance is usually provided and, in a few cases, an accreditation. In Belgium, for instance, the local union of architects and the Belgian Building Research Institute organise a compulsory one-day training on radon with credits for architecture students. In Ireland, radon remediation and prevention courses were developed in the past until 2016 in collaboration with the construction and the radon industry and will resume in 2023. The national RAP in Ireland calls to work in partnership with the relevant professional bodies to develop a continuing professional development module on radon. In the UK, training courses on radon mitigation are also available for interested contractors.

#### Guidelines and instructions on corrective actions

Some countries, like Belgium, focus their mitigation efforts on developing and publishing technical guidance on mitigation. Although the guidelines provide information on technical solutions to the house owners if high radon levels are found, the guidelines are country-specific for the prevailing national building and geological conditions. Therefore, most of the countries still need to develop their own evidence-based guidelines. In Ireland, as part of the RadoNorm project, the EPA, Wexford libraries and the Healthy Wexford group have been working with citizen scientists within the Wexford community who have found levels of radon in their homes above the reference level of 200 Bq/m3 and have not yet remediated to reduce their levels. For this, citizen scientists have helped co-design and test a “*Do it yourself*” (DIY) kit for mitigation together with EPA and a radon remediation contractor.

### Mitigation industry (contractor’s) perspectives

Little is known about contractors’ experience of running a radon mitigation business. In the following sections we present evidence for how contractors start and maintain a business, manage advertising, required competencies and facilitate homeowner engagement.

#### Getting started in the service

Two aspects appeared important to setting up services to provide radon mitigation to homes. One route to service provision arose from having existing business in radon and enough work, such as in a large government contract to justify the move into domestic radon mitigation. This route into radon contracting generally started with response to tender for schools, social housing or hospital contracts. A second route into service provision came from those who were working in existing companies where radon contracting was already present and who decided to leave to start their own radon mitigation services

“*I was providing concrete cutting services. So I have diamond tip the drills for, you know, drilling large holes… it was at the time that the government was doing the mitigation work in the schools. There’s money to be made and we had the men, we had the equipment*.” Interviewee 3.

#### Viability of radon mitigation services

A minimum viable business needs to have repeatable sales and momentum, which can be interpreted as actual or predicted turnover. None of the interviewees believed that a radon mitigation-only business was viable.

The contractors interviewed appeared to suggest that they normally completed 1 to 3 jobs per month for a local or regional company, and a maximum of 8 for a national company using subcontractors. All interviewees stated it was currently not possibly to run a domestic radon mitigation only business but that it would normally be a minor part of their main business.

“*There is no recurring business in the radon industry at the moment… so nobody in their right mind is gonna set up a company and assign overheads to it… when you just don’t know you’re gonna get the business… the only possibility where there’s a possibility of business happening is through contracted business… say social housing … where you can become part of reactive and planned maintenance of existing property stock.*” Interviewee 2.

The sales conversion rate is quite poor, which further undermines the viability of the business.

“*I would say for the general public our efforts are pretty poorly responded to. Everyone is seems so interested and everyone seems so grateful at that point of getting information out of you, to the point where even potentially purchasing a test kit. But the actual return, or the conversion of a high reading into mitigation is probably quite low.*” Interviewee 1.

Despite the campaigns raising radon awareness, demand for radon mitigation for some contractors has been consistently low for almost a decade.

“…*we probably do three or four jobs a month… after eight years… we’ve seen no real increase*.” Interviewee 1.

Given the low level of demand, one contractor stated that it was, “…*not learning how to install radon fans…*(*but*) *learn how to run a business and make it business viable in the very small segment of market*.” Interviewee 2.

Homeowners are reluctant to mitigate because of “*Cost, pure and simple*” Interviewee 4. but those who are likely to mitigate are, “*…people that have a disposable income*,” Interviewee 6.

Based on the above challenges, one contractor who refused to grant an interview had already left the radon business as there were not enough domestic jobs to justify continuing.

#### Advertising radon mitigation services

Given the issues noted with gaining customers, it was noteworthy that contractors typically do not advertise as the costs are not viable compared to the market reach: the customer base tends to be regional or even national. While some contractors will engage in awareness campaigns, they are dependent on mass media campaigns run by government or trade bodies such as the EPA in Ireland or UKRA.

“*If it’s not happening, I can’t make it happen, right? I’m completely reliant on others* (*mass communicators*) *to create a need for it*.” Interviewee 3.

Contractors maintain visibility to the public through being on national (e.g., EPA/UKRA) register, using Google Ads, and having their own web pages to provide information. None of the contractors used radio advertisements or flyers:

“*… maybe 9 or 10 years ago we put … flyers … into all the papers and the post office … the feedback wasn’t huge … we spent a bit of money in that … I have boxes of these brochures still out there anyway, I’d be slow to do that again.*” Interviewee 4.

Only one company actively used social media and blogs and previously hired a public relations company to manage social media for them.

Purchasing local advertising is only worth it if enough sales can be generated to cover the advertising costs and return a profit. Homeowners were reported to be more motivated to test after regional/national advertising and therefore look for contractors locally. However, one contractor highlighted that Google Ads are very “*congested*” now and may not be worth it as, “*spending absolute premium money just to get your ads on the first page does not make any sense you know because of the margins*.” In addition, when competing companies appear in a search the” *customer cannot discriminate between an expert and non-expert*.” Interviewee 2.

#### Motivation to provide services

Some contractors described their motivation to provide radon mitigation in terms of the health benefits, although the need for the service to be commerically viable was emphasised.

“*I just think about it from the health of just general health of people… we want our families to be healthy*.” Interviewee 2

“*I’d always go moral over over financial to be fair, within reason…* (*but*) *I’m still a businessman*.” Interviewee 4.

#### Regulation

Contractors noted that the building regulations for ventilation and radon are not well known: “…*building regulations for ventilation are based on minimum standards*…*there is so much ignorance actually in the trade relating to this… most installers out there do not know the regulations to the level that they should do for ventilation…*.” Interviewee 2.

“*…I can even speak to health and safety people in this area who were like* ‘*radon what’s radon?*.’” Interviewee 1.

One contractor (Interviewee 2) highlighted the problems caused by the failure to install ventilation equipment correctly “*I walk into properties and see they have been…not installed physically correctly, so they have used the wrong duct*” and “*we see more often than not…the electrical compliance side of those installations, people putting them in with on off switches…. because people turn them off and then and then suddenly there’s no ventilation*.”

### Residents’ perspectives

The barriers and facilitators indentified by the residents can be categorised into four broad categories: individual factors (risk perception, knowledge of how to mitigate and cost), inter-individual factors (trust), organisational factors (access to mitigation services) and governmental factors. It should be noted that some issues transcend these categories but are described here under these headings for simplicity of presentation.

#### Individual factors

- *Risk perception*

Residents who mitigated their dwellings in Ireland perceived radon as a high risk: the threat may affect them or their family members, especially children, which may have negative health consequences:

“*we have children as well, which we want to protect. So that’s why we went we better get it done*.” (IRE)

In contrast, non-mitigators noted that lung cancer is a complex issue, which cannot be explained solely by radon.

“*I’m not totally convinced of the of the need for it* (*mitigation*)*. I’m not convinced about the dangers of it* (*radon*)*. I don’t think I’ve ever come across anybody with lung cancer as a result of radon.*” (IRE)

The importance of communicating the level of risk in a way that motivates individuals was noted across groups:

“(risk communication)*… I think it’s important … The authorities and the companies don't show this sense of urgency*.” (BEL)

“*To inform and make people aware about radon risks.*” (SLOV)

- *Knowledge of how to mitigate*

A barrier to remediation arises from the lack of information on how to proceed:

“*Would need to know what to do step-by-step after receiving the measurement result. Who can help me?*.” (SLOV)

“*They do tests, but they’re long term tests and it, it uh, it gives you the average in the house but it doesn’t help to know how to fix the problem and where the real leaks are and stuff like that which is imperative to actually fix the problem*.” (BEL)

An interesting experience from one of the participant in Slovenia was shared during the focus group. The participant followed the mitigation guidelines published on the authorities’ internet page. The mitigation was not efficient since the radon was “*pushed*” from the ground floor to the highest floor (2,733 Bq/m^3^).

- *Cost*

Mitigation costs and costs for maintenance/running costs of mitigation were perceived as high by those in Ireland who did not mitigate.

“*A constant input of funds regarding ah to keep it* (*fan*) *running. And if you don’t do that or maintain it or the fan breaks down, you’re back to square one and you’re back to facing another cost to try and resolve the issue.*” (IRE)

The issue of cost arose in the focus groups in other countries too; therefore in order to facilitate greater levels of mitigation the costs should be affordable.

“*That costs related to remediation would not be too high.*” (SLOV)

“*It would be interesting to perhaps make the information accessible to people by telling them, um, it's perhaps not so difficult to remediate, nor so expensive.*” (BEL)

Some countries provide subventions to help home-dwellers and these financial supports can help overcome barriers associted with financial capacity to pay for the mitigation work. Being able to access such funding is a critical facilitator.

“*The council…paid practically all of it.*” (IRE)

### Inter-individual factors

#### Trust

Knowledge of the mitigation process will not translate into action if there is a lack of trust with the mitigation services. Non-mitigators have lower trust in the radon related information shared by authorities and contractors than mitigators.

“… *they had a vested interest maybe in selling radon barriers or something like that.*” (IRE)

In order to overcome the potential lack of trust, the integrity and independence of the information and service should be emphasised.

“*It will be someone who has both knowledge and expertise but who, ideally, is not necessarily a salesman from a company*.” (BEL)

“*… some people will have an issue with regards to a government agency recommending things to get done and people will be concerned…do they have a genuine reason behind that or are we just trying to meet certain targets to make the country look good?*.” (IRE)

In addition, a facilitator of mitigation is that the home dweller can trust the quality of the work performed to reduce radon levels in the home.

“*Because if I’m going to do something that’s going to cost me money, time, take steps, but if I’m not sure it’s going to be effective, …I’m going to drop it.*” (BEL)

“*That somebody would guarantee for quality of the remediation work.*” (SLOV)

Using personal testimonals and mass media to communicate the positive experiences of remediation, especially amongst those living in the same community, would faciliate greater trust and would encourage individuals to seek such services.

“*Personal testimonies of people during remediation*.” (SLOV)

“*More information on successful remediation and re-tests in mass media*.” (SLOV)

### Organisational factors

#### Access to mitigation services

Once a high level of radon has been detected, it is essential that information is provided to the home dwellers on accessing the mitigation services.

“*Would need to know what to do step-by-step after receiving the measurement result. Who can help me?*.” (SLOV)

In addition, having access to a sufficient number of appropriate mitigation services will facilitate increased level of mitigation for those with high levels of radon.

“*To have more remediation services available*.” (SLOV)

“*Well, we have, we just have our vision but, to give you an example, we have a list of companies in Luxembourg that should be able to deal with radon. We have contacted them all, the whole list, there is nobody who really has experience on it, but it’s on the list of experts. So, to make it work, to make people apply solutions, companies that are, uh, led to be professionals and experts that are not and they say it themselves that they are not. It’s a bit difficult.*” (BEL)

### Governmental/legal authority factors

The role of legal authories (e.g., national governmental, state /regional councils, etc) was noted in all focus groups. There was a general sense that as radon is a key health issue, the legal authorities should be more involved in pro-actively addressing radon; it should not be left to the individual.

“*If radon is a problem, than state should pay for tests and remediation*.” (IRE)

“*Will* (*would*) *the state co-finance the remediation?*.” (SLOV)

In Ireland and Belgium, those who mitigated suggested that the State should make radon mitigation compulsory, including radon in all relevant building legislation.

“*Central policy from government should have some carrots in it to entice people from all incomes to address a radon problem in their house, whether it be financial support, whether it be provision of a monitor, whether it be a certificate for your house before you sell it that it is radon free. Unless it’s highlighted from central government as a policy, people will not buy into it*.” (IRE)

“*I think it’s essential that there is a law that obliges, especially in new buildings, that there is an avoidance of radon risks by specific measures to be taken and that are verified by standardised tests or certified that it’s correct.*” (BEL)

### Integrated approach

The above sections have noted that barriers exist at individual, inter-interindividual, community, organisational and governmental levels. Many of the issues cut across these levels. Participants noted the fragmented nature of the mitigation process and appealed for an integrated approach from all stakeholders; such an approach would faciliate increased radon mitigation.

“*All experts need to work together and they need to communicate and increase awareness about measurements, remediation, general information about radon, about the process including about potential subventions*.” (SLOV)

## Discussion

The present study examined the perspectives of key stakeholders on the barriers and facilitators to the mitigation of the air pollutant radon. The key aspects identified by each stakeholder group are outlined separately before considering the novel insights that emerged by taking a multi-stakeholder lens.

The first facilitator, as recognised by the authorities, is a legal requirement for mitigation of privete dwellings in case of excedded levels of radon indoor as already highlighted previously (3). Generally, owners of public buildings with an exceeded radon concentration are legally obliged to mitigate the building, but in the case of private dwellings, mitigation is only advised. The second facilitator is evidence-based, and strategic communication and awareness campaigns, which are organised by authorities to motivate for behaviour change (testing and mitigation). The lack of such communication campaigns has been recognised by the Potsdam radon communication manifesto (16). Target groups for these campaigns vary among countries; howewer, they should be more targeted (17). Although increasing awarenes is necessary, it is not by itself sufficient to result in radon mitigation (23). The third facilitator is available financial support for residents to mitigate and simple processes to obtain the available grants. Although this finding confirms previous research (43), it should be noted that some countries provide financial support but there is a low uptake of available resources, including in one of the investigated countries (Ireland). The fourth facilitator is the connection with indoor air quality and energy saving programmess, which are currently not systematically linked. The lack of connection between radon risk management and other relevant policies was highglighted previosly as a barrier to mitigation (20). The authorities discussed how to consider radon in these programmess and the benefits of establishing links between different authorities for instance public health, spatial planning and nuclear safety authorities. Another facilitator is the accreditation or licence of radon mitigation contractors, which is not yet widely implemented. In the present paper accreditation and licensing is understood in the broadest sense of meaning any form of indication that the company has purposefully increased its competence in the field of radon mitigation measures, in particular by attending official courses, e.g., those organised by the regulatory authority. Authorities suggest that the main reason for the lack of motivation to become a radon mitigator is low profit, especially in the case of private dwellings. Given the perspective of the authorites regarding radon mitigation contractors motivation, a key strength of this paper is that we obtained data from contractors on their experiences of being in the radon mitigation industry.

To the best of our knowledge, this is the first study to examine radon mitigation from the perspective of the mitigation industry. Of interest, currently running a domestic home mitigation-only business is not feasible due to the low turnover of mitigation jobs. Consequently, all participants offered domestic mitigation work alongside other sales and services such as ventilation, conservation, commercial radon work or radon test sales, with mitigation as a minor service. Similar to the authorities’ perspective, the importance of awareness campaigns was noted by contractors: homeowners tend to respond to mass media campaigns and not *ad hoc* local advertising. Consequently, increasing the frequency of media campaigns and giving contractors prior notice to optimise their public relations strategies might produce higher mitigation rates.

Contractors typically do not advertise as the costs are high with poor sales conversion rates. Local advertising rates are high and only worth committing to if enough sales can be generated to cover the advertising costs and return a profit. None of the contractors used radio advertisements or flyers in recent years due to low response rates. Contractors suggested that homeowners found them during conveyancing, through either an online radon register or via an internet search. Membership of a professional register and having a good website are important. Cost also emerged as a facilitator for mitigation. As homeowners want to pay low costs for building work, contractors may choose the cheapest solution where minimum standards are specified.

Radon was associated with ventilation but the minimum standards for ventilation are often poorly understood by other professionals in decision-making roles. Those interviewed differentiated themselves from other tradespeople on their in-depth knowledge of the building regulations and the need for problem-solving for individual mitigation cases. As noted in the results, the experience of the resident who followed published radon mitigation guidance highlights the potential for inadequate ventilation solutions to inadvertently exacerbate the health risks of radon. This has implications for both improvements to regulations and training of radon mitigation installers. Support for this view is that recent current building regulations in the UK have been criticised for not being “fit for purpose” and poorly applied (44) and that radon-risk is further predicted to increase with different building designs, insulation, and air conditioning systems due to the impact of climate change (45, 46). This finding reflects the point made by the authorities regarding the value of integrating radon with energy saving programmes.

Lastly, this research looked at the perspective of residents concerning barriers and facilitators. Concerning the individual factors, risk perception varied among mitigators and non-mitigators, showing that risk perception can influences mitigation decisions, depending on a sense of urgency, personal relevance or even engagement with the health issue at stake. However, it should be noted that other research has not found a relationship between risk perception and mitigation; for example, people living in high radon affected areas find the risks of radon acceptable, despite being more concerned about these risks (47, 48). The question remains as to what variables differentiate those who mitigate from those who do not mitigate. One possible explanation of this discrepancy could be the health value and locus of control of the residents regarding radon. Residents with an internal locus of control might believe that they themselves have control over their health and therefore are more likely to take action, whereas residents with an external locus of control might believe their health is in the hands of others, or chance, and might avoid action (49). This could also be related to costs, as those who did not mitigate indicated that the burden of high costs was a barrier for mitigation.

When considering the inter-individual factors, trust is perceived as an important barrier to mitigate. Trust is related to the intentions of those who communicate about radon mitigations, but also about self-efficacy (confidence) of the residents to get the mitigation performed, and their perceptions of successful efficacy of the mitigation. The focus groups show that on the one hand side, guaranteeing results would increase their perceptions of its success, and on the other hand side, seeing testimonials of those who performed mitigation, would increase the homeowners’ self-efficacy to get the mitigation performed. Previous research showed that self-efficacy is a predictor of radon mitigation (50), which can be increased by modelling the behaviour through testimonials (51–53). The main facilitator in this regard would be to increase trust in the mitigators, the residents themselves, and the solution as an effective way to decrease radon levels in their homes.

Regarding the organisational factors, residents identified the need for clear information on the needed steps to mitigation, and on where to find the right services to facilitate this mitigation. This ties in with the barriers explained at the level of the mitigation industry, where the lack of viability of radon mitigation services is emphasized. A similar trend is visible when looking at the governmental factors, where the residents would prefer to see more government urgency and legislation regarding radon measures, however, only a minority of countries have these kinds of legislations in place. This summarises into the need for an integrated approach, highlighted by the residents, where all stakeholders should convey the same message, and support this message with appropriate action. Such an integrated approach would facilitate radon mitigation behaviour.

**Figure 1:**
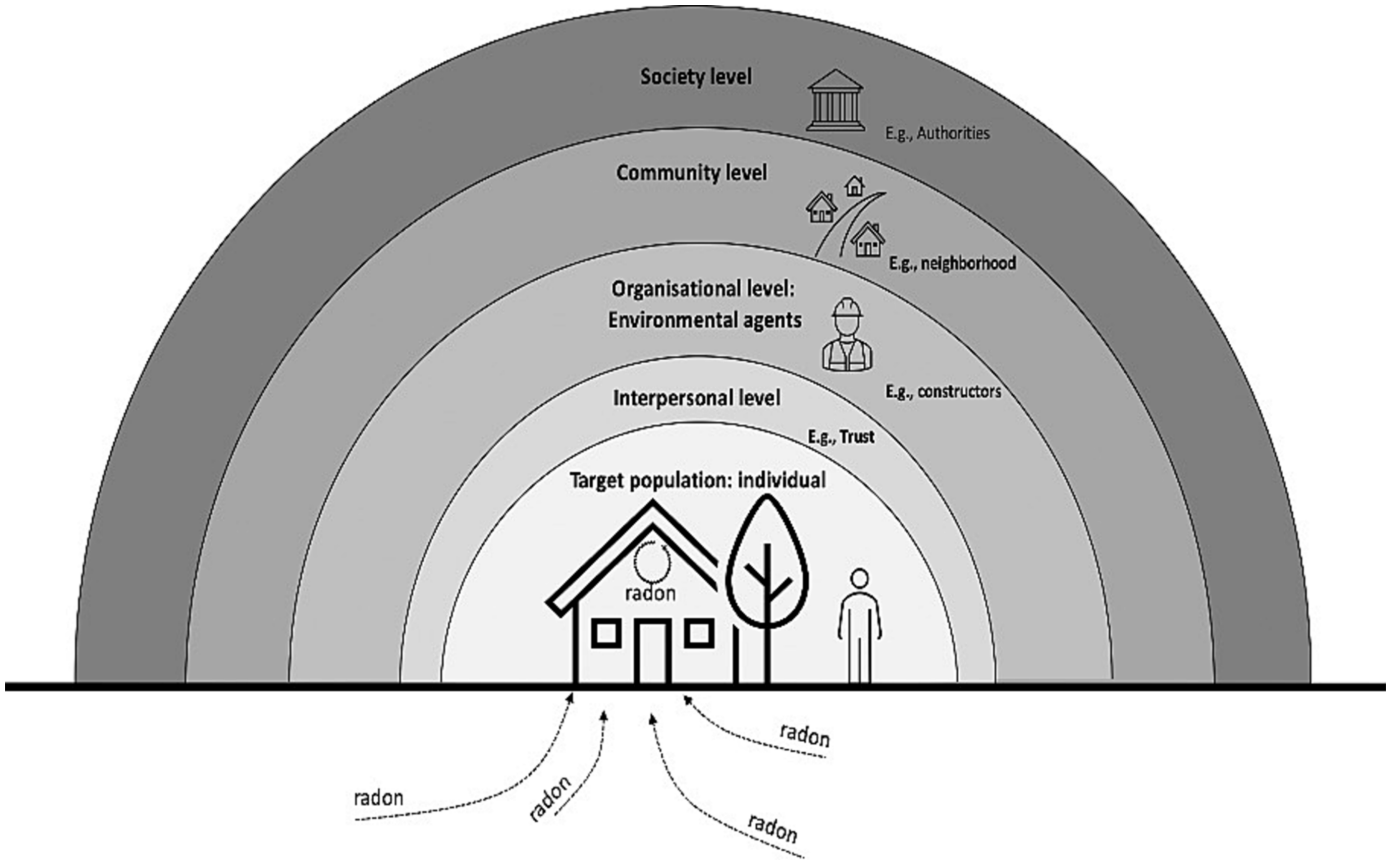
The individual is nested within various levels that influence radon mitigation rates.

Figure 1 illustrates the value of the psycho-social-environmental lens in helping understand the complexity of radon mitigation. Radon seeps into domestic dwellings and can permeate all levels of the dwelling. In the absence of a legal requirment to mitigate, the individual must decide whether to mitigate and this decision is influenced not just by the individuals knowledge and perception of risk from radon but also the cost, availability and accessibility of contractors to perform the mitigation; however individuals must have trust in the contractor’s qualifications and motivations for conducting the work. Examples of others in the individual’s community having mitigated also create trust in the need to mitigate and specific contractors. The present novel findings highlight the need to contextualise radon mitigation decision making in a complex system that requires facilitators to be present at multiple levels.

Based on the findings, Table 2 summarises the key radon mitigation facilitators identified by the stakeholders, the level(s) at which the facilitators operate, and the extent to which the facilitators are present. The absence of such facilitators creates a barrier to radon mitigation. The perception of the extent to which the facilitators exist in practice varies considerably and yet each contribute to the increased mitigation. Several facilitators were noted by all stakeholders: legal regulations/buildings code; evidence based and strategic awareness campaigns; accessible contractors, and affordable costs. In essence a campaign to make people aware of the health risks of radon is required, and for those with high levels of radon it must be easy to identify a trustworthy contractor and the cost should not be prohibitive. Financial support can be made available to support residents in undertaking the mitigation work. All of this needs to take place in the context of legal requirements to mitigate dwellings with an exceeded radon concentration, with clear policies connecting radon as an indoor pollutant with indoor air quality and energy saving programmes.

**Table 2:** Radon mitigaton facilitators identified by stakeholders.

Facilitators for mitigation [stakeholder level: legal (L), organisational (O) or individual (I)]	Recognised by the stakeholders group	Do the facilitators exist in practice according to the stakeholder group’s perception?
Legal requirements to mitigate dwellings with an exceeded radon concentration (L)	Authorities residents	No no
Building code (L)	Authorities contractors residents	Not sufficient not sufficient not sufficient
Evidence based and communication strategies focused on a behavioural change (L)	Authorites	Only in few countries
Awareness campaigns (L)	Authorities contactors residents	Yes yes, but not continuous yes
Connection with indoor air quality programmes (L)	Authorities	Not sufficient
Connection with energy saving programmes (L)	Authorities	Not sufficient: only in few countries
Accessible contractors (L, O, I)	Authorities contractors residents	Not sufficient not sufficient not sufficient
Sufficient demand for mitigation (O, I)	Contractors	Not suffciient
Affortable costs for mitigation (L, O, I)	Authorities contractors residents	No no no
Significant financial support for mitigation (L, O, I)	Authorities residents	Only in few countries only in few countries
Accreditation of radon mitigation contractors (L, O)	Authorities	Only in few countries
Education and training provided for contractors (L, O)	Authorities	Mainly yes
Trust in the mitigation process (I)	Residents	Not sufficient
Guidelines and instructions for mitigation available (L, O, I)	Authorities residents	Yes no
Risk perception (I)	Residents	Not sufficient

The present fiindings highlight that radon risk management is a complex “Wicked” problem; such problema are complex policy challenges that require a coordinated, cross-government response and where there is no obvious solution (54). For example, all stakeholders highlighted the importance of campaigns to increase awareness of radon to risk perception; yet even when awareness is high and radon is perceived as a risk, mitigation rates are low (19, 23). In addition, although all stakeholders noted the need for finiancial support for radon mitigiation, it should be noted that even where such supports are available, evidence indicates low levels of uptake. For example, free radon testing was offered in conjunction with a 50% grant towards necessary remedial work in a radon priority area in Ireland; however, only 20% took up this offer (42). Consequently, additional research is needed to understand why there is such a low uptake of the subventions, e.g., is it too difficult to access the subvention, it is not sufficient to meet the cost? The complexity of these issues means that, while the ultimate goal (e.g., radon mitigation) is clear, there is no obvious solution to achieve this goal. Rather, the solution may need to be arrived at incrementally from a psycho-social-environmental lens through an iterative process that involves stakeholders at all levels to create an integrated approach to radon mitigation. As noted, wicked environmental health problems are most likely to be addressed by the application of effective community health promotion skills, a sustained commitment to sound epidemiological science, the application of systems thinking, and transparent communication among all stakeholders (54). Communications about radon risk tend to be solely focused on awareness communications targeting the individual in radon priority areas to act; however, evidence indicates that, although necessary, these are not sufficient by themselves. Communication among all stakeholders requires clear, consistent and integrated communication and collaboration between all key radon responsible authorities.

Further research to examine the perspectives of residents and radon mitigation contractors in other countries is warranted. In addition, the perspectives of other key stakeholders (e.g., health care professionals, policy makers) across countries should be examined to further understand barriers and facilitators to radon mitigation.

## Conclusion

The article highlights that previous studies have only examined barriers to radon mitigation from the perspective of residents, but this study takes a more holistic approach by identifying psychological, social and enrivonmental facilitators and barriers to radon mitigation in four different countries for residents, responsible authorities and mitigation industry. The findings demonstrate the complexity of the radon mitigation process and suggest that interventions aimed at increasing mitigation should target groups beyond just residents, such as constructors, health professionals, and policy makers. Effective interventions should also utilise different media to reach specific audiences, but communication alone may not be enough to change behaviors if there are no contractors available to advise and assist residents. The study concludes that an integrated approach from radon mitigation policy to provision is necessary to effectively lower levels of this indoor air pollutant.

## Data availability statement

The raw data supporting the conclusions of this article will be made available by the authors, without undue reservation.

## Ethics statement

The studies involving humans were approved by Trinity College, Dublin (SPREC092021-04). The studies were conducted in accordance with the local legislation and institutional requirements. The participants provided their written informed consent to participate in this study.

## Author contributions

DH, TP, MM, GB, SA, and KR contributed to the conception and design of the study and conducted the data collection. DH, TP, MM, and GB contributed to the analyses. All authors contributed to the article and approved the submitted version.

## Funding

This project has received funding from the Euratom Research and Training Programme 2019–2020 RadoNorm under grant agreement No. 900009 and from the EU-RAP study under contract ENER/2020/NUCL/SI2.837814 (“*Review and evaluation of national radon action plans established in EU Member States according to the requirements in Council Directive 2013/59/Euratom*”).

## Conflict of interest

The authors declare that the research was conducted in the absence of any commercial or financial relationships that could be construed as a potential conflict of interest.

## Publisher’s note

All claims expressed in this article are solely those of the authors and do not necessarily represent those of their affiliated organizations, or those of the publisher, the editors and the reviewers. Any product that may be evaluated in this article, or claim that may be made by its manufacturer, is not guaranteed or endorsed by the publisher.

## Footnotes

^1^ https://www.seai.ie/home-energy/home-upgrades/guide-to-upgrades/
